# The effects of melatonin in the treatment of acute brachial plexus compression injury in rats

**DOI:** 10.3389/fneur.2023.1111101

**Published:** 2023-03-01

**Authors:** Xigong Li, Jing Fu, Haiying Zhou, Yanzhao Dong, Ahmad Alhaskawi, Zewei Wang, Jingtian Lai, Chengjun Yao, Sohaib Hasan Abdullah Ezzi, Vishnu Goutham Kota, Mohamed Hasan Abdulla Hasan Abdulla, Ming Guan, Xianfeng Lou, Hui Lu

**Affiliations:** ^1^Department of Orthopedics, The First Affiliated Hospital, College of Medicine, Zhejiang University, Hangzhou, Zhejiang, China; ^2^Department of Stomatology, Affiliated Hangzhou Xixi Hospital, Zhejiang University School of Medicine, Hangzhou, China; ^3^Department of Clinical Medicine, Zhejiang University School of Medicine, Hangzhou, Zhejiang, China; ^4^Department of Orthopaedics of the 3rd Xiangya Hospital, Central South University, Changsha, Hunan, China; ^5^Alibaba-Zhejiang University Joint Research Center of Future Digital Healthcare, Zhejiang University, Hangzhou, Zhejiang, China

**Keywords:** acute brachial plexus compression injury, melatonin, rat model, myelin protein zero, nerve regeneration

## Abstract

**Introduction:**

Brachial plexus injury (BPI) is one of the most destructive peripheral nerve injuries and there is still a lack of effective treatment.

**Methods:**

This study was conducted to evaluate the effects of melatonin in the treatment of acute brachial plexus compression injury in rats using histopathological, histomorphometric, immunohistochemical and electrophysiological methods. Forty-eight adult male Sprague Dawley rats were randomly allocated into three groups: sham, melatonin and vehicle groups. The brachial plexus compression injury model was performed by a vascular clamp. Melatonin group received intraperitoneal injection of melatonin at doses of 10 mg/kg for 21 days after crush injury. The conduction velocity and amplitude of compound muscle action potential (CAMP) in the regenerated nerve, and nerve histomorphometry, as well as levels of myelin protein zero (P0) protein of the crush region were assessed.

**Results:**

Compared with the vehicle group, the melatonin group which reported significant increased CMAP conduction velocity and amplitude also showed thicker myelin sheath and lower levels of P0 protein.

**Discussion:**

Our results suggest that melatonin effectively promotes nerve regeneration and improves the function of damaged nerves. Melatonin treatment is a promising strategy for the treatment of acute brachial plexus compression injury.

## 1. Introduction

Brachial plexus injury (BPI) is one of the most destructive peripheral nerve injuries, which can result in notable motorsensory deficits (1–3). Despite neurosurgical techniques are available to repair the lesion, function restoration of BPI remains unsatisfactory in clinical practice (2). The outcomes after nerve reconstruction are adversely affected not only by mechanical injury, also by several secondary factors such as inflammatory responses, oxidative stress and regenerative misdirection (4). Several recent studies have focused on the promising approaches involving therapeutic management of the secondary cascade resulting from BPI (4, 5).

Melatonin, the main hormone of the pineal gland, daily regulates circadian rhythms (6, 7). Various formulations of melatonin have been patented and used in the treatment of sleep dysfunction. Some previous studies show that melatonin can regulate various physiological functions including anti-inflammation, free radical scavenging and anti-oxidative properties (7). Due to these biological properties, melatonin has been used as potential pharmacotherapy for traumatic events in the central nerve system. Recently, it has been demonstrated that melatonin has have beneficial effects on peripheral nerve repair in models of sciatic nerve injury (8–13).

In this study, we evaluated the potential neuroprotective roles of melatonin in brachial plexus compression injury models of rats.

## 2. Materials and methods

A total of 48 adult male Sprague Dawley (SD) rats, weighing about 250 g, were obtained from experimental animal center of Zhejiang University. The animal experiment was approved by the Zhejiang University's Animal Experimentation Ethics Committee. All rats were housed in a room with constant and appropriate temperature and humidity and are subjected to a 12-h diurnal cycle. Drinking water and standard laboratory feed were available in sufficient quantities. They were allowed to acclimatize for 1 week prior to the experiments. Efforts were made to minimize animal suffering and to reduce the number of animals used during the experiments.

**Figure 1:**
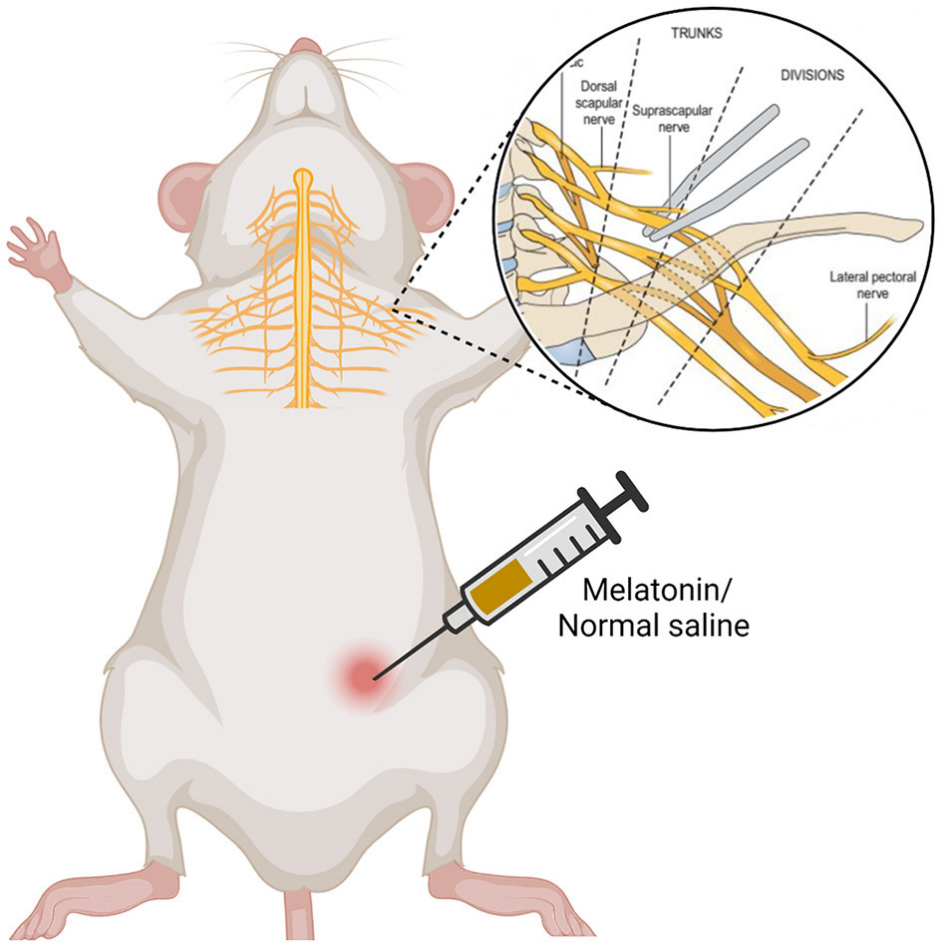
The modeling diagram.

The animals were randomly divided into 3 groups (*n* = 16 each group): sham, vehicle and melatonin groups. Sham group had no operation on the brachial plexus nerve, instead, they just underwent skin incision and suturing. In melatonin and vehicle groups, all surgical procedures were performed after anesthesia with an intraperitoneal injection of xylazine and ketamine (10 and 80 mg/kg). The left brachial plexus was identified and exposed using an operating microscope. Distal to the suprascapular nerve branch, the upper trunk of the brachial plexus were crushed by a mosquito vascular clamp (Shanghai Medical Instruments Corp. Ltd.) that was fixed to the maximun extent for 30 s. Afterwards, the incision was closed by using 4.0 silk sutures. Melatonin group received intraperitoneal injection of melatonin at doses of 10 mg/kg for 21 days after injury. Normal saline was injected intraperitoneally in vehicle group (Figure 1).

By 7 and 14 days after surgery, 6 rats were anesthetized and perfused transcardially with 0.5% glutaraldehyde in 0.1 M phosphate buffer. Short 5 mm segments of the brachial plexus were collected. The specimens were fixed in 2.5% phosphate-buffered glutaraldehyde solution (PBS) for 1 h at room temperature and then at 4°C, until processed. Tissue paraffin blocks were fabricated by post-fixing specimens in 1% OsO4 in PBS, dehydrating in a graded series of alcohol and propylene oxide, embedding in resin, and then polymerizing at 60°C. Transverse semi-thin sections (1 mm) were obtained using an ultramicrotome, and were respectively stained with Fast blue (Abcam, UK), silver (Amresco, USA) and H&E staining (Abcam, UK). By 7 days after surgery, ultrathin sections (70 nm) of nerve samples were stained with uranyl acetate and citrate and were evaluated by JEM1400 transmission electron microscope (JEOL Ltd., Tokyo, Japan).

Three weeks after surgery, the injured sites of the brachial plexus were excised and lysed, and resolved by 10% sodium dodecyl sulfate polyacrylamide gel electrophoresis, and then transferred to a polyvinylidene difluoride membrane, blocked with 5% non-fat dry milk. Subsequently, the membrane was incubated with mouse polyclonal antibody to P0 (1:1,000; Santa Cruz Biotechnology, USA) and mouse monoclonal antibody to GAPDH (1:5,000; Santa Cruz Biotechnology). After washing with 0.1 M Tris buffered saline (Ph 7.2) containing 0.1% Tween-20 (TBST) for three times (10 min for each), the membranes were incubated with rabbit anti-mouse IgG (1:5,000; Santa Cruz Biotechnology), for 2 h at room temperature. Band optical density values were determined using Gel-Pro Analyzer Software, version 4.0 (Media Cybernetics, USA).

Three weeks after surgery, 4 rats were anesthetized and the left brachial plexus was isolated. Bipolar stimulating electrodes were placed near the injury site while the bipolar recording electrode was placed in the upper limb muscle. The amplitude and latency of compound muscle action potential (CMAP) were recorded with an electromyogram instrument. The bipolar stimulating electrodes were used to stimulate the regenerated nerves and the bipolar recording electrodes inserted into the muscle were to record electrical activity through a digital MYTO electromyograph machine (Esaote, Genoa, Italy).

All pathological manifestations, Fast blue staining, HE staining, silver staining or transmission electron microscopic images, were evaluated by a pathologist who was blinded to this study. Quantitative variables were described using means and standard deviations (SD), and compared using *t*-tests. The level of significance was set to *p* < 0.05. All analyses were done in SPSS 23.0.

## 3. Results

**Figure 2:**
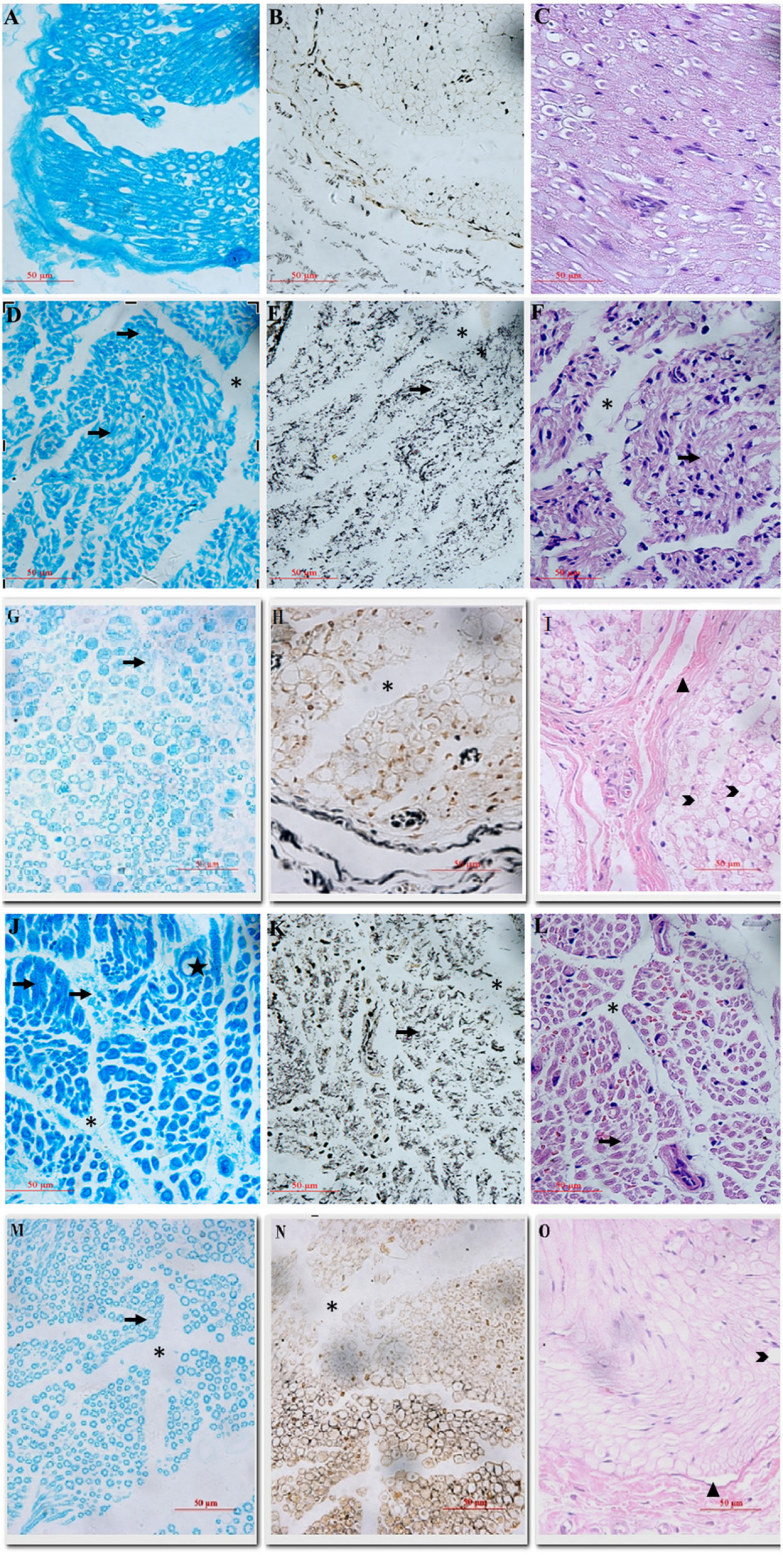
The general views of histological sections stained by Fast blue, silver and H&E. **(A–C)** Sham group: well-arranged and distributed nerve fibers were observed; **(D–I)** vehicle groups at 7 and 14 days: typical features of peripheral nerve injury after 7 days could be seen in **(D–F)**, including myelin loss with Wallerian degeneration (arrow), and endoneurial edema (*). **(G–I)** A further decrease in nerve fiber density, disruption of fiber arrangement, and a marked increase in fibrosis (triangle) and axonal vacuolization (arrowhead) at day 14. **(J–O)** Melatonin groups at 7 and 14 days: compared with vehicle group, less axonal degeneration and vacuolization, as well as fibrosis were observed, with more regenerating axon clusters (star) and the nerve fibers were are neatly aligned.

**Figure 3:**
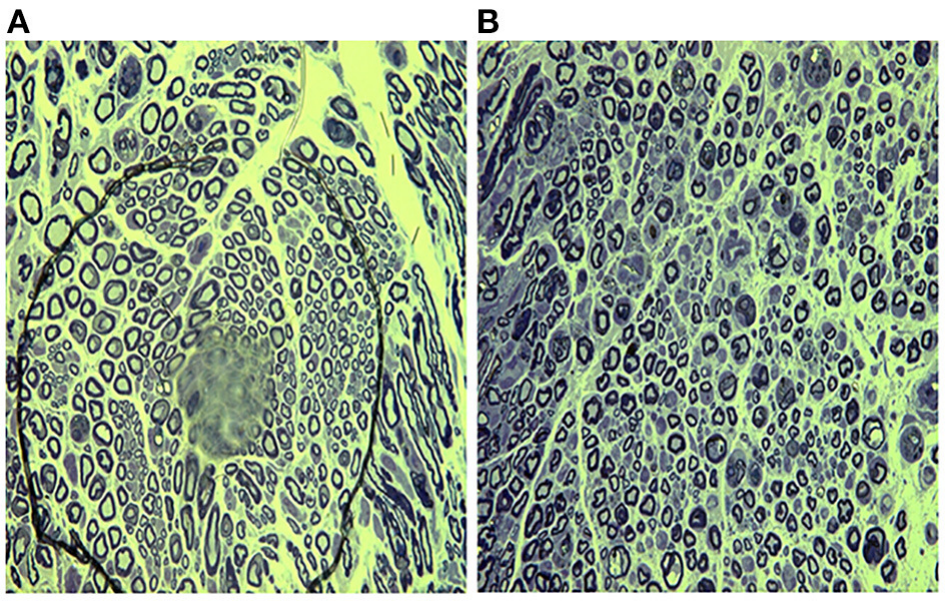
Transmission electron micrographs showing the ultrastructure of brachial plexus day 7 after injury. **(A)** Vehicle group; **(B)** melatonin group. Nerves in the melatonin group showed thicker myelin sheaths and narrower endoneural gaps compared to the vehicle group. Scale bar = 5 mm.

Well-arranged and distributed nerve fibers were observed in sham group (Figures 2A–C). On the contrary, the typical characteristics of peripheral nerve injury were present in vehicle group on day 7, including axonal degeneration, myelin abnormalities, and endoneurial edema (Figures 2D–F). By day 14, the density of nerve fibers was further reduced, and the axonal degeneration was increased markedly. More axons had collapsed and the number of Schwann's cells increased (Figures 2G–I). Compared with vehicle group, less axonal degeneration and vacuolization were observed in melatonin group (Figures 2J–O). And the vehicle group showed slightly more increase in endoneural space than the melatonin group. Similarly, the transmission electron microscope analysis showed there was a significant difference in myelin sheath thickness and endoneural space between the vehicle and melatonin group (Figures 3A, B).

**Figure 4:**
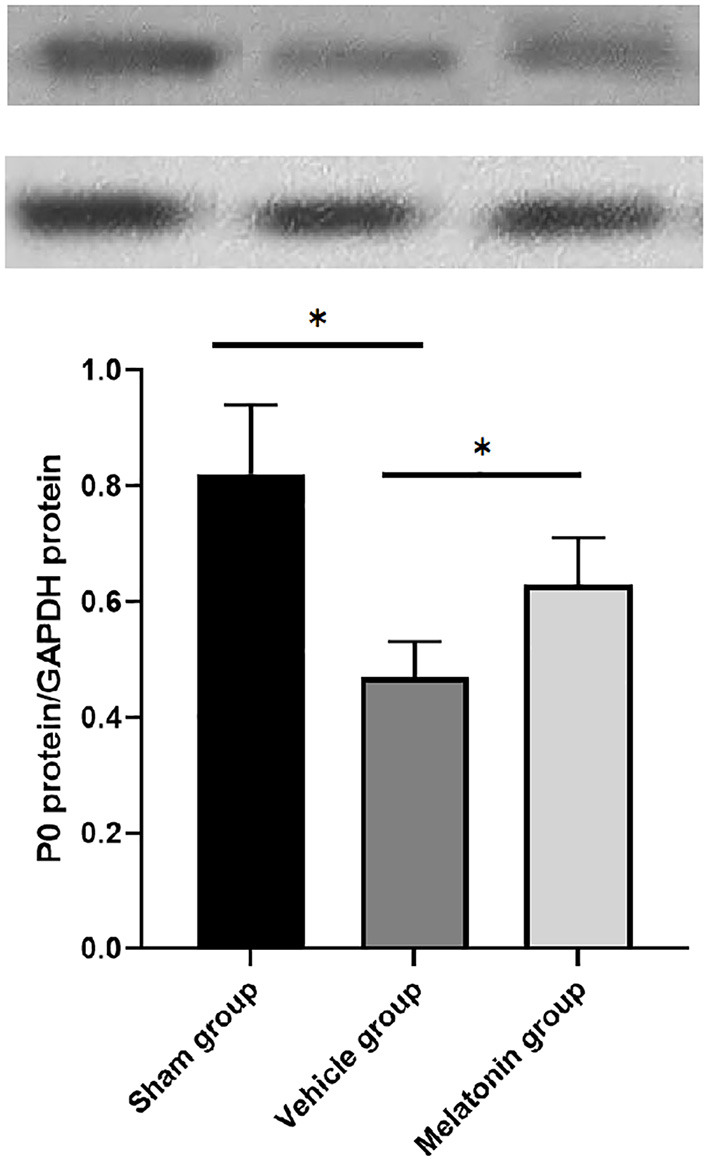
P0 protein/GAPDH protein levels were assessed using western blotting in the injured sites of rats in all 3 groups, 3 weeks after injury. *p*-value ^*^ < 0.05.

The levels of P0 protein at 3 weeks after surgery in all three groups were shown in Figure 4. The levels of P0 protein were significantly decreased in vehicle group compared with the sham group (*P* < 0.05). Whereas, the P0 protein levels in the melatonin group, although slightly lower than the sham group, were significantly higher compared with vehicle group (*P* < 0.05).

Table 1 showed the results of electrophysiological assessment at 3 weeks after surgery in all three groups. The CMAP amplitude was reduced markedly (*p* < 0.05), while the CMAP latency was prolonged significantly (*p* < 0.05) in vehicle group compared with that in Sham group. Moreover, melatonin-treated group displayed distinctly higher CMAP amplitude (*p* < 0.05) and markedly shorter CMAP latency (*p* < 0.05) compared with vehicle group.

**Table 1:** The results of electrophysiological evaluation in all three groups.

**Group**	**Sham**	**Vehicle**	**Melatonin**
CMAP latency (ms)	2.21 ± 0.08	3.57 ± 0.16^*^	3.08 ± 0.06^#^
CMAP amplitude (Mv)	11.68 ± 0.8	1.92 ± 0.24^*^	5.34 ± 0.14^#^

* *P* < 0.05 for the sham compared with vehicle group;

# *P* < 0.05 for the melatonin compared with vehicle group.

## 4. Discussion

Peripheral nerve injury of the upper limb is a common and extremely inconvenient clinical disease. The main mechanisms of its occurrence are compression, trauma, peripheral nerve tumor, inflammation, neuronal degeneration, and radiation exposure (14–16). The brachial plexus is not only the most complicated structure in the peripheral nervous system, but highly susceptible to trauma, or may be damaged secondary to lesions of adjacent structures (17). The present study demonstrated continuous treatment with melatonin can significantly increase CMAP conduction velocity, enhance myelin sheath thickness and at the same time, increase P0 protein levels in the regenerated brachial plexus nerve after injury compared with vehicle treatment. These results suggest that melatonin can effectively promotes nerve regeneration and improves the function of damaged nerves. Melatonin treatment is a promising strategy for the treatment of acute brachial plexus compression injury.

There are some basic pathological changes in the early stage of peripheral nerve injuries, including axonal degeneration, myelin abnormalities, and endoneurial edema (4). And severe peripheral nerve injuries can finally lead to the Wallerian degeneration of the distal segment of the nerve, which means rupture of the axonal membrane, degradation of cytoskeletal components and lysis of myelin sheaths (4, 18). Therefore, several pharmacological agents have been investigated to facilitate myelination and functional recovery after peripheral nerve injury.

In the process of peripheral nerve repair, Schwann cells proliferate and construct the myelin sheath to enhance axonal regeneration from proximal to distal (18). P0, the major peripheral nervous system myelin protein, belongs to the immunoglobulin supergene family of membrane proteins and can mediate homotypic adhesion (19, 20). P0 is considered as a fundamental structural component of peripheral nervous system myelin, and is directly or indirectly involved in the regulation of myelin gene expression and myelin morphogenesis (20). And the levels of P0 expression are related to Schwann cell myelination in peripheral nerve repair (21). The experimental results of the present study showed that P0 protein levels in the myelin sheath were increased after melatonin treatment, from which it can be inferred that melatonin treatment triggered Schwann cell proliferation and myelination, thus promoting the repair and regeneration of damaged nerves. Other histomorphological findings corroborate this supposition. The electrophysiological assessment showed that injured nerve after melatonin treatment can provides lower latency recordings and higher amplitude measures, which indicated a higher number of normally functioning axons in the melatonin treatment group. And histological observation manifested the presence but not nimiety of Wallerian degeneration, more regenerating axon clusters, higher myelin thickness and nerve fiber density, more orderly nerve fiber arrangement in the melatonin group, all of which support the promotion of melatonin for nerve regeneration.

Melatonin is a hormone secreted by the pineal gland of brain and produce a marked effect on the regulation of sleep and circadian rhythm (1, 2). Previous studies demonstrated melatonin has neuroprotective effects in the treatment of peripheral nerve injury (8–13, 22). Melatonin treatment can markedly increase the number of axons and thickness of myelin sheath in rat models of sciatic nerve injury (8–13, 22). And structural protection of the myelin lamellae can be preserved following repeated low dosage of melatonin administration (13). Even a single injection of high-dose melatonin can act to protect myelin sheath, prevent axonal loss, and accelerate functional recovery of the injured sciatic nerve (12). Pan et al. (11) illustrated that melatonin might promote peripheral nerve regeneration by improving the proliferation and migration of Schwann cells through the Shh signaling pathway after sciatic nerve injury. Moreover, recently simultaneous use of melatonin and chondroitin sulfate ABC synergistically promoted nerve regeneration in the brachial plexus nerve-root avulsion model (23). All these experimental results were consistent with our findings.

Overall, this study strengthens the idea that melatonin promoted axonal regeneration after brachial plexus compression injury, which may provide a promising therapeutic strategy for peripheral nerve injury. However, our study had some limitations. Firstly, histomorphometric changes with experimental injury and healing response were assessed only by histology and immunohistochemistry. Secondly, we did not conduct functional evaluation. In addition, further experiments should be conducted on the proper dosage and possible side effects.

## Data availability statement

The original contributions presented in the study are included in the article/supplementary material, further inquiries can be directed to the corresponding author.

## Ethics statement

The animal study was reviewed and approved by the First Affiliated Hospital, College of Medicine, Zhejiang University.

## Author contributions

HL and XL designed the study. XL, HZ, and MG performed data collection. AA, JF, YD, and CY analyzed the results. JL, ZW, VK, MA, and SE drafted the manuscript. All authors have read and approved the final manuscript.
